# Chromosome-level genome assembly of the freshwater bivalve *Anodonta woodiana*

**DOI:** 10.1038/s41597-025-05078-6

**Published:** 2025-05-02

**Authors:** Xiubao Chen, Tao Jiang, Junren Xue, Mengying Gu, Meiyi Wang, Kai Liu

**Affiliations:** 1 Freshwater Fisheries Research Center, Chinese Academy of Fishery Sciences, Wuxi, 214081 China; 2 Wuxi Fisheries College, Nanjing Agricultural University, Wuxi, 214081 China; 3 College of Marine Science and Technology and Environment, Dalian Ocean University, Dalian, 116023 China

**Keywords:** Genome, Molecular biology

## Abstract

The freshwater bivalve *Anodonta* (*Sinanodonta*) *woodiana* originated in the Yangtze River basin of China and is now widely distributed in Asia, Europe, North America, and Africa. This species has important economic and ecological value. Using Illumina, PacBio, and Hi-C technology, a high-quality chromosome-level genome of *A. woodiana* was assembled. The genome size was 2.80 Gb, with a contig N50 of 4.01 Mb and a scaffold N50 of 143.34 Mb. In total, 1609 contigs, accounting for 99.57% of the total assembled genome, were anchored into 19 chromosomes. In total, 1.51 Gb repeat sequences were annotated and 44,785 protein-coding genes were predicted. This study is the first to reveal the genome of *A. woodiana* and the genus *Anodonta*, which will effectively contribute to investigations of this species’ biology, molecular mechanisms in response to environmental stress, and resource management.

## Background & Summary

**Fig. 1:**
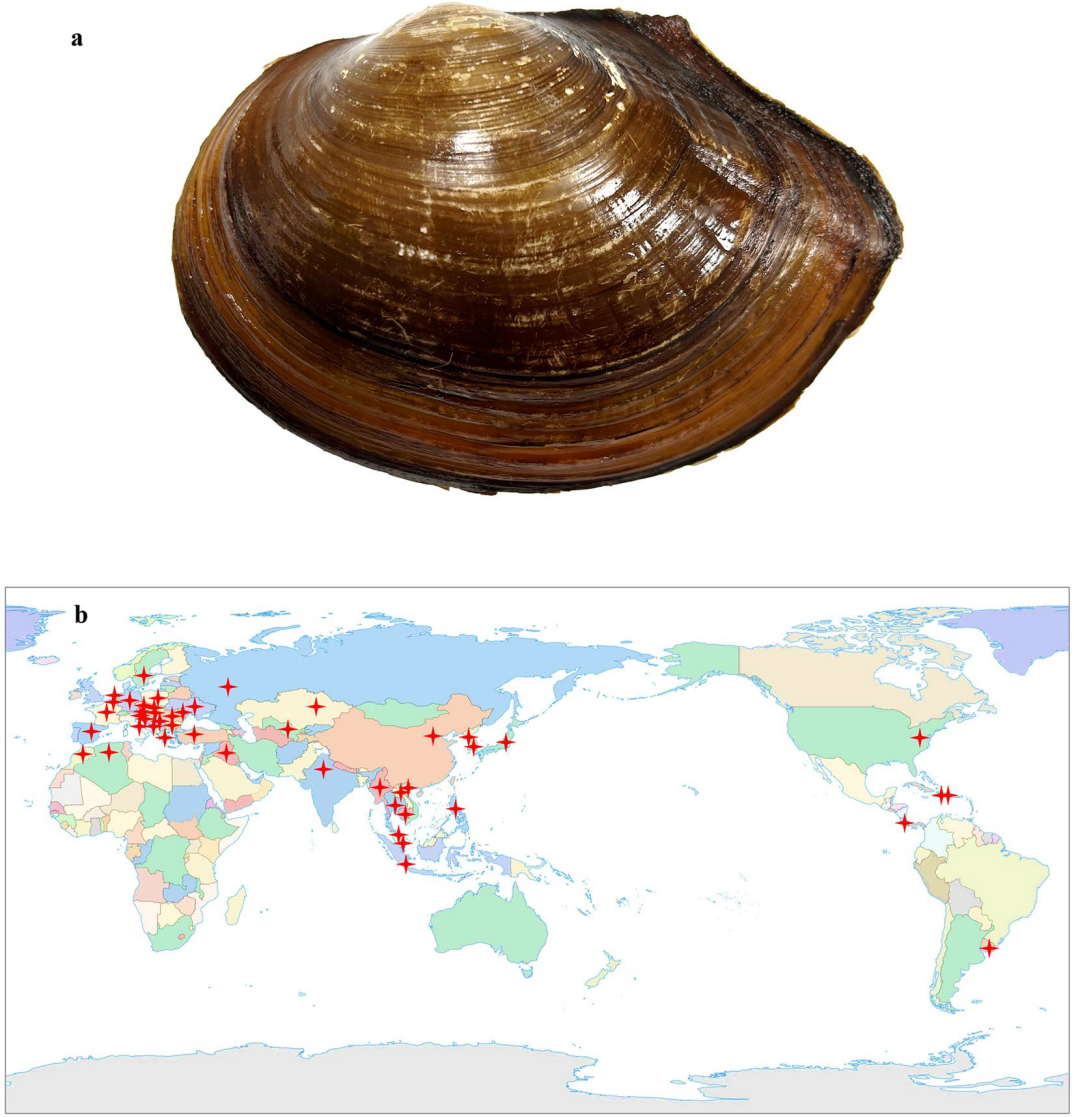
*Anodonta woodiana* (**a**) and its global distribution (**b**). The cross indicates the countries with *A. woodiana* distribution.

The freshwater bivalve *Anodonta* (*Sinanodonta*) *woodiana* (Fig. 1a), commonly known as the Chinese pond mussel or Chinese mussel, originates from the Yangtze River basin in China^1–3^. Over the decades, this species has spread to 46 countries in Asia, Europe, North America, and Africa (Fig. 1b) via host fishes^4–6^. Moreover, it can live in a variety of freshwater environments, such as rivers, lakes, reservoirs, and ponds.

*Anodonta woodiana* has high economic value, as it is used for pearl cultivation in China^4^ and Indonesia^7^. In China and Poland, it has been developed as a high-protein food^4,8,9^, and in Italy, it is used as a new alternative ingredient for rainbow trout (*Oncorhynchus mykiss*) feed^10^. Furthermore, it contains active antitumor substances and can be used to make antitumor drugs^11^. Additionally, *A. woodiana* has important ecological value. In freshwater ecosystems, it acts as an “ecosystem engineer”^4^. Owing to its widespread distribution, narrow range, and high accumulation of pollutants, but low metabolism, it was identified as a unique bioindicator for the “Freshwater Mussel Watch” programs^4,12^, and thus, it is widely used for monitoring and evaluating organic (e.g., pesticides) and inorganic (e.g., heavy metals) contamination in freshwater ecosystems^13–15^.

In its native country, natural *A. woodiana* resources have declined sharply^16^. For example, the resources of this species in Poyang Lake, the largest freshwater lake of China, have declined by 70% over a 26-year period (1981–2007)^17^. Heavy metal (e.g., Cu) pollution was proved to be a key causal factor in many water areas^18^. Therefore, investigating the toxic effects of heavy metals on *A. woodiana* has becoming a research hotspot^19–24^. However, owing to the lack of reference genomic information for *A. woodiana*, the study of related molecular mechanisms has been very limited.

Genomic information plays a crucial and fundamental role in investigating biological characteristics^25^, environmental toxicology^26^, and resource conservation^27^. Here, we completed sequencing and assembly of the *A. woodiana* genome using Illumina Novaseq X Plus sequencing, PacBio long-read sequencing (PacBio), and high-throughput chromosome conformation capture (Hi-C) technologies. Genome annotation includes repetitive sequence annotation, non-coding RNA annotation, and mRNA prediction and the associated functional annotation. This study is the first to reveal the genome of *A. woodiana* and the genus *Anodonta*, which will effectively contribute to investigations of this species’ biology, molecular mechanisms in response to environmental stress, and resource management.

## Methods

### Ethics declaration

The study protocol was approved by the Ethics Committee of the Freshwater Fisheries Research Center, Chinese Academy of Fisheries Sciences (protocol code: LAECFFRC-2022-06-13).

### Sample collection and sequencing

A female *A. woodiana* (age, 4 years; shell length, 13.3 cm) specimen was collected on February 20, 2024, from the Freshwater Fisheries Research Center of the Chinese Academy of Fishery Sciences (Wuxi, China). It was cultured in aerated tap water for 3 weeks, with a complete water change every 2 days, during which time it was kept in an aerated environment and not fed to empty the gills. The gills were dissected for genomic DNA extraction^28^. DNA sequencing methods were as described previously^26^. Briefly, genomic DNA was extracted using the SDS method. DNA libraries were constructed by fragmenting the DNA, followed by damage repair, end repair, the ligation of junction sequences, enzymatic digestion, and purification of the fragmented DNA. The HiFi sequencing mode of the PacBio Sequel II platform (PacBio, USA) was applied for sequencing. A Hi-C library was constructed through cell cross-linking, enzymatic digestion, end repair, cyclization, and DNA purification and capture. After the library was qualified, high-throughput sequencing was performed using the Illumina Novaseq 6000 platform (Illumina, USA). RNA was extracted with TRIzol reagent. RNA-seq libraries were constructed via mRNA enrichment, reverse transcription, PCR amplification, damage and end repair, and sequencing junction ligation using the SQK-LSK 109 (ONT) kit. Full-length transcriptome sequencing was performed using the Illumina Novaseq 6000 platform. In total, 104.94 Gb HiFi reads, 233.54 Gb Hi-C data, and 6.12 Gb RNA raw reads were obtained (Table 1).

**Table 1:** Statistical analysis of the sequencing data of the *Anodonta woodiana* genome.

Type	Number of Reads	Number of Bases (bp)	Mean Read Length (bp)	N50 Read Length (bp)	GC (%)	Q20 (%)	Q30 (%)
HiFi	6,679,350	104,941,281,552	15,711	16,333	34.75	—	—
RNA	4,552,216	6,116,210,151	1,343	1,790	40.86	—	—
Hi-C	781,304,810	233,536,069,893	150	150	35.60	99.35	97.68

### Estimation of genome size

**Fig. 2:**
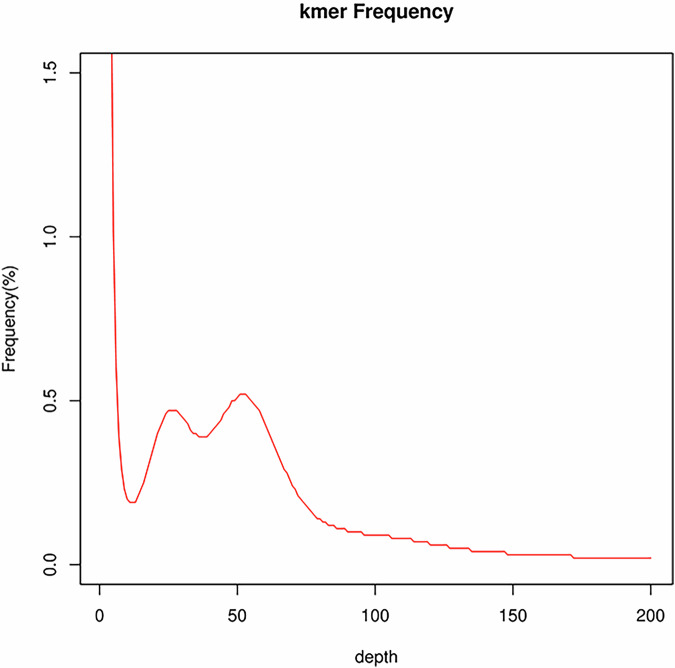
Frequency distribution of the K-mer of the sample.

The K-mer method^29^ was used to assess the genome size, heterozygosity, and repeat sequence content in *A. woodiana*. We performed a k-mer (k = 17) frequency distribution analysis using 135.2 Gb of Illumina clean data (Fig. 2). For the genomic characteristic determination, de novo genome studies often use a smaller k-mer size, mostly 17^30^. This selection is driven by two primary rationales, as follows: (1) the total k-mer space (4^17^ = 16 Gb) is sufficiently larger than the genome size of most common genomes and thus has the ability to store all the k-mers derived from the genomes; and (2) using a larger k-mer size will result in more erroneous k-mers caused by sequencing errors and then decrease the efficiency of this method^30^. In total, 120,713,639,752 k-mers with a depth of 52 were obtained. The genome size was estimated to be 2.12 Gb, showing a moderate heterozygosity of 1.15% and a high repeat rate of 71.63%.

### Genome assembly

PacBio HiFi sequencing is currently the primary sequencing method for genome assembly^26,31^ because of the high-accuracy reads (>99%) obtained through the cyclic sequencing of the same DNA sequence while ensuring long read lengths (10–20 kb). Illumina Hi-C sequences were used to assist in the assembly^26,31^. For HiFi data, we used Hifiasm (v0.19.9) software^32^ with default parameters for assembly, and the obtained primary contigs were adjusted for the subsequent analysis. For Hi-C data, the contigs were first clustered using Juicer (v2.0) and 3D-DNA software^33^ to determine the closeness of the association between the contigs; then, the interactions between contig pairs were converted into a specified binary file (i.e.,.hic file) using Juicertools (v3.0) software; thereafter, the contigs were analyzed using Juicebox (v2.15.07) software^34^ to manually correct the sequenced and oriented contigs to obtain the final scaffold assembly results.

**Fig. 3:**
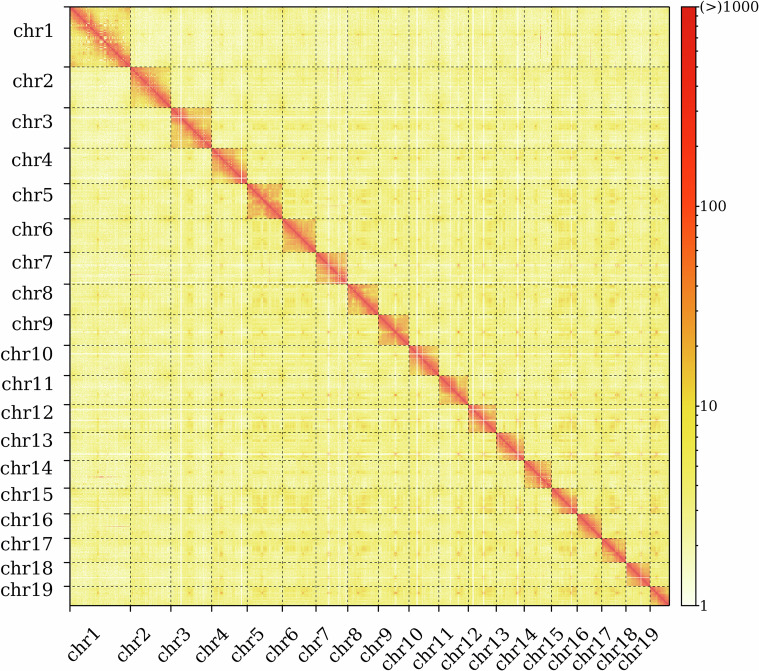
Heatmap of 19 chromosome interactions based on Hi-C data.

**Fig. 4:**
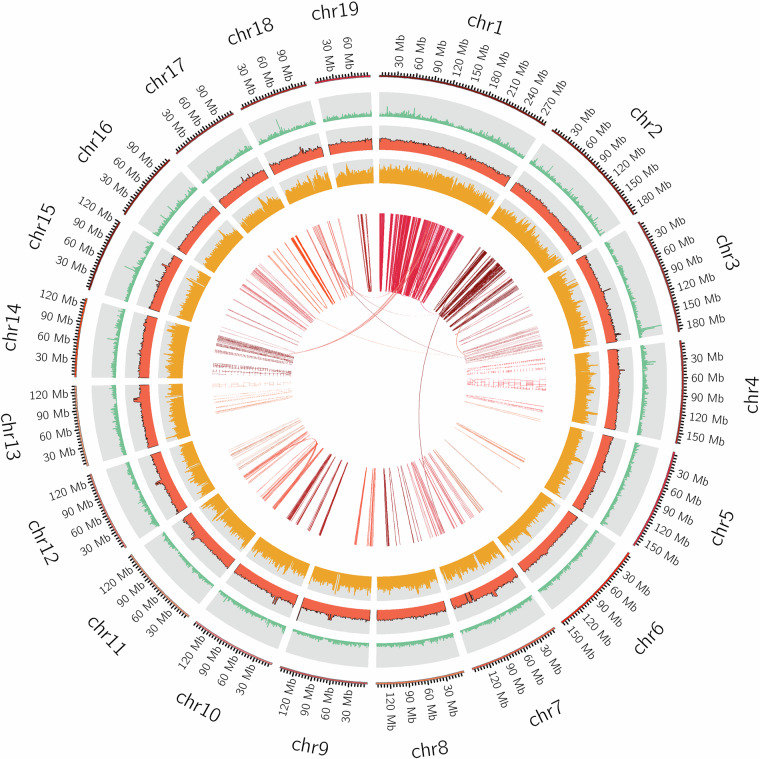
Genome characteristics of *Anodonta woodiana*. The first, second, third, and fourth circles (from outside to inside) indicate the different chromosome sequences, gene density, GC content, and repetitive sequence content, respectively, and the center-most connecting lines indicate genes with collinearity.

Based on HiFi and Hi-C sequencing data, a high-quality chromosome-level genome of *A. woodiana* was assembled. The genome size was 2.80 Gb, and there was notable discrepancy (32.1%) between the assembled and estimated genome sizes. A previous study demonstrated that a high repeat rate and heterozygosity can lead to a significant underestimation of the genome size based on a K-mer analysis^35^. For example, in the black sea urchin (*Arbacia lixula*), the K-mer-estimated genome size was 31.1% lower than the assembled genome size owing to its high repeat rate (41.84%) and high heterozygosity (4.40%)^35^. This suggests that the substantial underestimation of the predicted genome size in *A. woodiana* is likely attributable to its high repeat rate. The contig N50 and scaffold N50 of the genome were 4.01 Mb and 143.34 Mb, respectively (Table 2). In total, 1609 contigs, accounting for 99.57% of the total assembled genome, were anchored into 19 chromosomes (Table 3; Fig. 3). Additionally, the genome characteristics of *A. woodiana* mapped with circos (v0.69-6) are shown in Fig. 4.

**Table 2:** Statistical analysis of genome assembly of *Anodonta woodiana*.

Parameter	Scaffold	Contig
N50	143,343,525	4,007,853
L50	8	205
N75	130,511,731	2,017,200
L75	13	450
N90	112,825,520	982,779
L90	17	748
GC (%)	35	34.66
Total length (bp)	2,804,107,447	2,803,948,447
Longest length (bp)	281,845,975	21,281,537

**Table 3:** Statistical analysis of chromosome assembly.

Chromosome	Length (bp)	Contig number
chr1	281,845,975	190
chr2	190,407,915	126
chr3	189,452,844	73
chr4	166674347	113
chr5	164,761,221	78
chr6	156,443,777	75
chr7	148,733,821	82
chr8	143343525	61
chr9	143,114,612	74
chr10	140592063	87
chr11	136,715,478	73
chr12	131,363,673	72
chr13	130,511,731	83
chr14	128307984	80
chr15	120,459,701	64
chr16	114,841,230	64
chr17	112,825,520	83
chr18	112683785	88
chr19	89,977,916	43
chrUn	1050329	7
Chr	2,803,057,118	1609
Total	2,804,107,447	1616

### Genome annotation

Repeat sequences can be categorized into scattered and tandem repeat sequences. These were first predicted for the genome, de novo, using RepeatModeler (v2.0.1)^36^, and then, the predicted results were merged with the RepBase database (http://www.girinst.org/repbase)^37^. Further, RepeatMasker (v4.1.0)^36^ was used to predict the repetitive sequences of the genome. In total, 1.51 Gb of repetitive sequences was identified, accounting for 53.96% of the total genome (Table 4). Particularly, DNA transposons were the most important repetitive sequences, accounting for 24.51% of the total genome (Table 4).

**Table 4:** Statistical analysis of the prediction results of repeated sequences in *Anodonta woodiana*.

Type	De novo + Repbase (bp)	In genome (%)
DNA	687,362,445	24.51
LINE	193,134,583	6.89
SINE	101,090,680	3.61
LTR	81,485,282	2.91
Satellite	12,620,553	0.45
Other	30,985,696	1.11
Unknown	406,365,438	14.49
Total	1,513,044,677	53.96

Non-coding RNA includes a variety of RNAs with known functions, such as rRNA, tRNA, snRNA, and miRNA, as well as RNA with unknown functions. The prediction of non-coding RNA consisted of three parts, specifically the prediction of rRNA sequences using Barrnap (v0.9)^26,38^, tRNA sequences using tRNAscan (v2.0.0)^39^, and non-coding RNAs based on a search of the Rfam database (ftp://ftp.ebi.ac.uk/pub/databases/Rfam/) using Infernal (v1.1.3)^40^. In total, 471 rRNA, 64,020 tRNA, 626 snRNA, 688 miRNA, 6,515 Cis-reg, and 42 ribozyme sequences were identified (Table 5).

**Table 5:** Non-coding RNA annotation of the *Anodonta woodiana* genome.

Type	Number	Average length (bp)	Total length (bp)	In genome (%)
**rRNA (total)**	471	179	84,601	0.0030
18S	6	1,814	10,884	0.0004
28S	6	3,369	20,217	0.0007
5.8S	7	152	1,064	0.0000
5S	452	116	52,436	0.0019
**tRNA**	64,020	73	4,708,270	0.1679
**snRNA (total)**	626	158	99,375	0.0035
CD-box	101	108	10,975	0.0004
HACA-box	20	217	4,347	0.0002
scaRNA	1	125	125	0.0000
splicing	504	166	83,928	0.0030
**miRNA**	688	95	65,412	0.0023
**Cis-reg (total)**	6,515	46	303,000	0.0108
leader	9	85	766	0.0000
other	6,506	46	302,234	0.0108
**ribozyme**	42	246	10,349	0.0004

The mRNA prediction included ab initio, homology, and RNA-Seq-based predictions. Ab initio prediction was performed using Augustus (v3.3.3) and GlimmerHMM (v3.0.4)^41^ software. For homology annotation, protein sequences of the freshwater mussels *Margaritifera margaritifera*^42^, *Potamilus streckersoni*^28^, *Sinosolenaia oleivora*^31^, *Unio delphinus*^43^, and *Unio pictorum*^44^ were aligned to the genome sequence using GeMoMa (v1.9)^45^. RNA-seq data were reconstructed from transcripts obtained using StringTie (v2.1.3)^46^, and then, coding frames were predicted using TransDecoder (v5.1.0). The multiple obtained datasets were integrated using EVidenceModeler (v1.1.1)^47^, and finally, the integrated data were updated using PASA (v2.5.2) to add UTR regions and discover new transcripts. After removing 3,380 isoforms, 44,785 protein-coding genes were predicted (Table 6), which is comparable to the number of protein-coding genes reported in many freshwater bivalve species, such as *P. streckersoni* (41,065)^28^, *U. delphinus* (44,382)^43^, and *U. pictorum* (46,138)^44^. The nucleic acid sequences of the longest transcripts of the predicted genes were compared with those of the NR, Swiss-Prot, GO, COG, KOG, and KEGG databases using BLAST (v2.10.1+)^48^ software, and the amino acid sequences were compared with those of the Pfam database using HMMER (v3.2.1)^49^ software to obtain the annotation information of the predicted genes. In total, 36,687 genes were annotated, of which 15,907 genes were between 300 and 1,000 bp in length, and 18,907 genes were longer than 1,000 bp (Table 7).

**Table 6:** Statistical analysis of gene predictions in the *Anodonta woodiana* genome.

Methods	Gene set	Gene number	Average gene length (bp)	Average CDS length (bp)	Average exon number per gene	Average exon length (bp)	Average intron length (bp)
Ab initio	AUGUSTUS	59,206	17,844	1,168	4.48	261	4,796
	GlimmerHMM	223,917	11,252	458	3.23	142	4,848
Homology	*Margaritifera margaritifera*	40,052	16,235	1,267	5.89	215	3,060
	*Potamilus streckersoni*	37,922	15,489	1,229	5.59	220	3,109
	*Sinosolenaia oleivora*	29,017	15,894	1,307	5.71	229	3,097
	*Unio delphinus*	43,463	15,664	1,230	5.78	213	3,021
	*Unio pictorum*	43,561	15,700	1,238	5.78	214	3,024
RNA-seq	TransDecoder	15,944	42,653	1,576	9.06	422	4,754
Integration	EVM	44,989	17,802	1,251	5.51	227	3,670
Final set	PASA	44,785	21,026	1,275	5.81	322	3,866

**Table 7:** Functional annotations of predicted genes.

Annotation database	Annotated number	300 ≤ Length (bp) ≤ 1000	Length (bp) ≥ 1000
COG	8,622	2,598	5,917
GO	16,945	5,278	11,301
KEGG	29,658	11,854	16,810
KOG	18,683	5,891	12,367
Pfam	27,312	10,217	16,305
Swissprot	18,281	5,723	12,157
NR	35,583	15,270	18,663
Total	36,687	15,907	18,907

## Data Records

The genomic Illumina sequencing, PacBio sequencing, and RNA-Seq were uploaded to the NCBI Sequence Read Archive (SRA) database under the accession number SRP547275^50^, with the BioProject accession number PRJNA1189286 and genome accession JBJQND000000000^51^. The genome assembly and annotation files are available in the Figshare database^52^.

## Technical Validation

### Evaluating the quality of the DNA and RNA

Extracted DNA/RNA was assessed for its concentration using a Nanodrop 2000 spectrophotometer (Thermo Fisher Scientific, San Jose, CA, USA) and a Qubit 3.0 fluorometer (Invitrogen, Carlsbad, CA, USA), and its integrity was examined using agarose gel electrophoresis (Electrophoresis apparatus: Tanon, Model: EPS600).

### Evaluating the quality of the genome assembly and annotation

The completeness of the genome assembly and annotation was assessed using BUSCO software (v5.2.1)^53^. The reference BUSCO database was the Metazoan dataset (metazoa_odb10)^54^. Among the 954 BUSCO groups searched, 931 (97.6%) of the complete BUSCO genes were found in the assembly (Table 8), and 944 (98.9%) complete BUSCO genes were detected in the annotation (Table 8). The BUSCO results indicate the high-quality of the assembly and annotation.

**Table 8:** Completeness of the genome assembly and annotation based on BUSCO.

Type	Assembly		Annotation	
	Gene number	Percentage (%)	Gene number	Percentage (%)
Complete (C)	931	97.6	944	98.9
Complete and single-copy (S)	854	89.5	859	90.0
Complete and duplicated (D)	77	8.1	85	8.9
Fragmented (F)	13	1.4	6	0.6
Missing (M)	10	1.0	5	0.5
Total	954	100	954	100

## Data Availability

The manuscript did not use custom code to generate or process the data described.

## References

[1] Kondakov, A. V. *et al*. DNA analysis of a non-native lineage of *Sinanodonta woodiana* species complex (Bivalvia: Unionidae) from Middle Asia supports the Chinese origin of the European invaders. *Zootaxa.* **4462**, 511–522 (2018). 30313456 10.11646/zootaxa.4462.4.4

[2] Konečný, A. *et al*. Modelling the invasion history of *Sinanodonta woodiana* in Europe: Tracking the routes of a sedentary aquatic invader with mobile parasitic larvae. *Evol Appl.* **11**, 1975–1989 (2018). 30459842 10.1111/eva.12700 PMC6231479

[3] Douda, K. *et al*. Review of the globally invasive freshwater mussels in the genus *Sinanodonta* Modell, 1945. *Hydrobiologia.* **852**, 1243–1273 (2025).

[4] Chen, X., Yang, J., Liu, H. & Jiang, T. Freshwater Mussel Watch: An innovative approach for interpretations of aquatic pollution and toxicology. *J Lake Sci.* **33**, 11–27 (2021).

[5] Mabrouki, Y. & Taybi, A. F. The first record of the invasive Chinese pond mussel *Sinanodonta woodiana* (Lea, 1834) (Bivalvia: Unionidae) in the African continent. *Nat Croat.* **31**, 393–398 (2022).

[6] Bensaâd-Bendjedid, L., Telailia, S., Alliouche, F., Touati, H. & Ladjama, I. First record of the occurrence of the Chinese pond mussel *Sinanodonta woodiana* (Lea, 1834) (Bivalvia: Unionidae) in African freshwaters: Oubeira Lake, Algeria. *Turk J Zool.* **47**, 94–102 (2023).

[7] Rahayu, S. Y. S., Solihin, D. D., Manalu, W. & Affandi, R. Nucleus pearl coating process of freshwater mussel *Anodonta woodiana* (Unionidae). *HAYATI J Biosci.* **20**, 24–30 (2013).

[8] Konieczny, P. *et al*. DSC and electrophoretic studies on protein denaturation of *Anodonta woodiana* (Lea, 1834). *J Therm Anal Calorim.* **126**, 69–75 (2016).

[9] Stangierski, J. *et al*. Effect of washing on the quality of surimi-like preparation obtained from soft tissue of freshwater mussel *Sinanodonta woodiana* (Lea, 1834). *J Aquat Food Prod T.* **27**, 961–974 (2018).

[10] Sicuro, B. *et al*. Freshwater mussel meal as new alternative ingredient for rainbow trout (*Oncorhynchus mykiss*) feeds: Growth performance and histomorphological analyses. *Aquacult Int.* **32**, 431–445 (2024).

[11] Liu, J. *et al*. Antitumor activities of liposome-incorporated aqueous extracts of *Anodonta woodiana* (Lea, 1834). *Eur Food Res Technol.* **227**, 919–924 (2008).

[12] Yang, J., Harino, H., Liu, H. & Miyazaki, N. Monitoring the organotin contamination in the Taihu Lake of China by bivalve mussel *Anodonta woodiana*. *Bull Environ Contam Toxicol.* **81**, 164–168 (2008). 18511994 10.1007/s00128-008-9464-z

[13] Bian, X., Liu, H., Gan, J., Li, R. & Yang, J. HCH and DDT residues in bivalves *Anodonta woodiana* from the Taihu Lake, China. *Arch Environ Contam Toxicol.* **56**, 67–76 (2009). 18465166 10.1007/s00244-008-9173-y

[14] Chen, X., Su, Y., Liu, H. & Yang, J. Active biomonitoring of metals with cultured *Anodonta woodiana*: A case study in the Taihu Lake, China. *Bull Environ Contam Toxicol.* **102**, 198–203 (2019). 30374584 10.1007/s00128-018-2482-6

[15] Yan, M., Chen, X., Xue, J., Liu, H. & Yang, J. Reconstruction of historical metal backgrounds in lacustrine environments of China using an *Anodonta woodiana* “specimen bank”. *Bull Environ Contam Toxicol.* **112**, 78 (2024). 38796607 10.1007/s00128-024-03906-w

[16] Chen, X. *et al*. Host fish suitability for freshwater bivalve *Anodonta woodiana* breeding programs. *Fishes.* **7**, 329 (2022).

[17] Liu, Y. J. Resource status and reproductive traits of freshwater bivalves in the Poyang Lake. Master’s Thesis (Nanchang Univ., 2008).

[18] Bian, B., Zhou, Y. & Fang, B. B. Distribution of heavy metals and benthic macroinvertebrates: Impacts from typical inflow river sediments in the Taihu Basin, China. *Ecol Indic.* **69**, 348–359 (2016).

[19] Liu, H. *et al*. The valve movement response of three freshwater mussels *Corbicula fluminea* Müller 1774, *Hyriopsis cumingii* Lea 1852, and *Anodonta woodiana* Lea 1834 exposed to copper. *Hydrobiologia.* **770**, 1–13 (2016).

[20] Xia, X. *et al*. Characterization of calmodulin in the clam *Anodonta woodiana*: Differential expressions in response to environmental Ca^2+^ and Cd^2+^. *Turk J Biochem.* **43**, 403–416 (2018).

[21] Chen, X. *et al*. Cadmium bioaccumulation and distribution in the freshwater bivalve *Anodonta woodiana* exposed to environmentally relevant Cd levels. *Sci Total Environ.* **791**, 148289 (2021). 34126494 10.1016/j.scitotenv.2021.148289

[22] Li, Y. Q., Chen, C. M., Liu, N. & Wang, L. Cadmium-induced ultrastructural changes and apoptosis in the gill of freshwater mussel *Anodonta woodiana*. *Environ Sci Pollut Res.* **29**, 23338–23351 (2022). 10.1007/s11356-021-16877-w 34811609

[23] Chen, X., Liu, H., Liber, K., Jiang, T. & Yang, J. Copper-induced ionoregulatory disturbance, histopathology, and transcriptome responses in freshwater mussel (*Anodonta woodiana*) gills. *Fishes.* **8**, 368 (2023).

[24] Yan, M. *et al*. Copper induces cytotoxicity in freshwater bivalve *Anodonta woodiana* hemocytes. *Chemosphere.* **362**, 142595 (2024). 38866330 10.1016/j.chemosphere.2024.142595

[25] Bai, Z. *et al*. The first high-quality genome assembly of freshwater pearl mussel *Sinohyriopsis cumingii*: New insights into pearl biomineralization. *Int J Mol Sci.* **25**, 3146 (2024). 38542120 10.3390/ijms25063146 PMC10969987

[26] Zhao, H. *et al*. A high-quality chromosome-level genome assembly of the topmouth culter (*Culter alburnus* Basilewsky, 1855). *Sci Data.* **11**, 910 (2024). 39174585 10.1038/s41597-024-03657-7 PMC11341867

[27] Tan, M. P. *et al*. Applications of next-generation sequencing technologies and computational tools in molecular evolution and aquatic animals conservation studies: A short review. *Evol Bioinform.* **15**, 1176934319892284 (2019). 10.1177/1176934319892284 PMC6896124 31839703

[28] Smith, C. H. A high-quality reference genome for a parasitic bivalve with doubly uniparental inheritance (Bivalvia: Unionida). *Genome Biol Evol.* **13**, evab029 (2021). 33570560 10.1093/gbe/evab029 PMC7937423

[29] Liu, B. *et al*. Estimation of genomic characteristics by analyzing k-mer frequency in de novo genome projects. *Quant Biol.* **35**, 62–67 (2018).

[30] Wang, H. *et al*. Estimation of genome size using k-mer frequencies from corrected long reads. *arXiv preprint arXiv 2003*, 11817 (2020).

[31] Ma, X. *et al*. Chromosome-level genome assembly of the freshwater mussel *Sinosolenaia oleivora* (Heude, 1877). *Sci Data.* **11**, 606 (2024). 38851789 10.1038/s41597-024-03451-5 PMC11162450

[32] Cheng, H., Concepcion, G. T., Feng, X., Zhang, H. & Li, H. Haplotype-resolved de novo assembly using phased assembly graphs with hifiasm. *Nat Methods.* **18**, 170–175 (2021). 33526886 10.1038/s41592-020-01056-5 PMC7961889

[33] Dudchenko, O. *et al*. De novo assembly of the *Aedes aegypti* genome using Hi-C yields chromosome-length scaffolds. *Science.* **356**, 92–95 (2017). 28336562 10.1126/science.aal3327 PMC5635820

[34] Robinson, J. T. *et al*. Juicebox.js provides a cloud-based visualization system for Hi-C data. *Cell Syst.* **6**, 256–258 (2018). 29428417 10.1016/j.cels.2018.01.001 PMC6047755

[35] Galià-Camps, C. *et al*. Chromosome-level genome assembly and annotation of the black sea urchin *Arbacia lixula* (Linnaeus, 1758). *DNA Res.* **31**, dsae020 (2024). 38908014 10.1093/dnares/dsae020 PMC11310861

[36] Tarailo‐Graovac, M. & Chen, N. Using RepeatMasker to identify repetitive elements in genomic sequences. *Curr Protoc Bioinformatics.* **25**, 4.10.11–14.10.14 (2009). 10.1002/0471250953.bi0410s25 19274634

[37] Bao, W., Kojima, K. K. & Kohany, O. Repbase update, a database of repetitive elements in eukaryotic genomes. *Mobile DNA.* **6**, 11 (2015). 26045719 10.1186/s13100-015-0041-9 PMC4455052

[38] Seemann, T. & Booth, T. GitHub - tseemann/barrnap: microscope: Bacterial ribosomal RNA predictor. *GitHub* https://github.com/tseemann/barrnap (2019).

[39] Chan, P. P., Lin, B. Y., Mak, A. J. & Lowe, T. M. tRNAscan-SE 2.0: improved detection and functional classification of transfer RNA genes. *Nucleic Acids Res.* **49**, 9077–9096 (2021). 34417604 10.1093/nar/gkab688 PMC8450103

[40] Nawrocki, E. P. & Eddy, S. R. Infernal 1.1: 100-fold faster RNA homology searches. *Bioinformatics.* **29**, 2933–2935 (2013). 24008419 10.1093/bioinformatics/btt509 PMC3810854

[41] Majoros, W. H., Pertea, M. & Salzberg, S. L. TigrScan and GlimmerHMM: Two open-source ab initio eukaryotic gene-finders. *Bioinformatics.* **20**, 2878–2879 (2004). 15145805 10.1093/bioinformatics/bth315

[42] Gomes-dos-Santos, A. *et al*. The Crown Pearl: A draft genome assembly of the European freshwater pearl mussel *Margaritifera margaritifera* (Linnaeus, 1758). *DNA Res.* **28**, dsab002 (2021). 33755103 10.1093/dnares/dsab002 PMC8088596

[43] Gomes-dos-Santos, A. *et al*. PacBio Hi-Fi genome assembly of the Iberian dolphin freshwater mussel *Unio delphinus* Spengler, 1793. *Sci Data.* **10**, 340 (2023). 37264040 10.1038/s41597-023-02251-7 PMC10235117

[44] Gomes-dos-Santos, A. *et al*. A PacBio Hi-Fi genome assembly of the painter’s mussel *Unio pictorum* (Linnaeus, 1758). *Genome Biol Evol.* **15**, evad116 (2023). 37341534 10.1093/gbe/evad116 PMC10329264

[45] Keilwagen, J., Hartung, F. & Grau, J. GeMoMa: Homology-based gene prediction utilizing intron position conservation and RNA-seq data. *Methods Mol Biol.* **1962**, 161–177 (2019). 31020559 10.1007/978-1-4939-9173-0_9

[46] Pertea, M. *et al*. StringTie enables improved reconstruction of a transcriptome from RNA-seq reads. *Nat. Biotechnol.* **33**, 290–295 (2015). 25690850 10.1038/nbt.3122 PMC4643835

[47] Haas, B. J. *et al*. Automated eukaryotic gene structure annotation using EVidenceModeler and the Program to Assemble Spliced Alignments. *Genome Biol.* **9**, R7 (2008). 18190707 10.1186/gb-2008-9-1-r7 PMC2395244

[48] Altschul, S. F., Gish, W., Miller, W., Myers, E. W. & Lipman, D. J. Basic local alignment search tool. *J Mol Biol.* **215**, 403–410 (1990). 2231712 10.1016/S0022-2836(05)80360-2

[49] Eddy, S. R. A new generation of homology search tools based on probabilistic inference. *Genome Inform.* **23**, 205–211 (2009). 20180275

[50] *NCBI Sequence Read Archive* https://identifiers.org/ncbi/insdc.sra:SRP547275 (2025).

[51] Chen, X. Sinanodonta woodiana isolate MN2024, whole genome shotgun sequencing project. *Genbank.* https://identifiers.org/ncbi/insdc:JBJQND000000000.1 (2024).

[52] Chen, X. Chromosome-level genome assembly of the freshwater bivalve *Anodonta woodiana*. *figshare*10.6084/m9.figshare.28004591 (2024). 10.1038/s41597-025-05078-6 PMC12048480 40316565

[53] Simão, F. A., Waterhouse, R. M., Ioannidis, P., Kriventseva, E. V. & Zdobnov, E. M. BUSCO: Assessing genome assembly and annotation completeness with single-copy orthologs. *Bioinformatics.* **31**, 3210–3212 (2015). 26059717 10.1093/bioinformatics/btv351

[54] Sukumaran, S. *et al*. The chromosome level genome assembly of the Asian green mussel, *Perna viridis*. *Sci Data.* **11**, 930 (2024). 39198463 10.1038/s41597-024-03802-2 PMC11358141

